# Decrease Rate of the Renal Diameter in Chronic Hemodialysis Patients

**DOI:** 10.5402/2013/521949

**Published:** 2013-05-22

**Authors:** Teiichiro Aoyagi, Masaaki Tachibana, Shinji Naganuma

**Affiliations:** ^1^Department of Urology, Tokyo Medical University Ibaraki Medical Center, 3-20-1 Chuo Ami, Ibaraki, Inashiki 300-0395, Japan; ^2^Department of Urology, Tokyo Medical University, Tokyo, Japan; ^3^Naganuma Clinic, Chiba, Japan

## Abstract

We here present the results of ultrasonographic (US) evaluations on the alteration of renal diameter of chronic HD patients. Of 109 outpatient HD patients who had neither severe acquired cystic disease of the kidney nor hereditary polycystic kidney disease, we performed US two or three times to measure their maximum renal diameter (mean of both kidneys), and the yearly alteration rate was calculated. The average interval of the two measurements was 35.9 months, and the average HD duration from the HD induction to the first measurement was 29.5 months. The average decrease rate of renal diameter was 4.34 ± 0.4 (SE) mm/year. No statistical difference was seen on the decrease rate in relation to gender, age and original disease (among three groups, glomerulonephritis and IgA nephropathy, diabetes, and others including hypertension). However, the decrease rate was large when the first measurement was close to the induction of hemodialysis, suggesting that the alteration rate reduced according to the hemodialysis vintage (5.3 ± 0.8 mm/year, first measurement not more than 10 months after induction of HD and 1.5 ± 1.6 mm/year, first measurement more than 80 months after induction of HD). Renal diameter decreased approximately 4.3 mm each year, and the decrease rate slowed as the length of time on dialysis increased.

## 1. Introduction

It is well known that the kidney size of the patient undergoing chronic hemodialysis (HD) gradually decreases [1]. However, only a few reports are there on the study of the actual decrease rate [2–4]. On the other hand, scheduled ultrasonographic (US) examination of kidney after induction of HD is important to check the progression of acquired cystic disease of kidney (ACDK) and renal cancer which may arise in ACDK [5, 6]. We examined the renal longest diameter of chronic HD patient at the scheduled US kidney checkup [7], calculated the alteration rate of the size in the same patients, and studied the difference of the alteration rate among the factors such as gender, age, and original diseases.

## 2. Subjects and Methods

**Figure 1:**
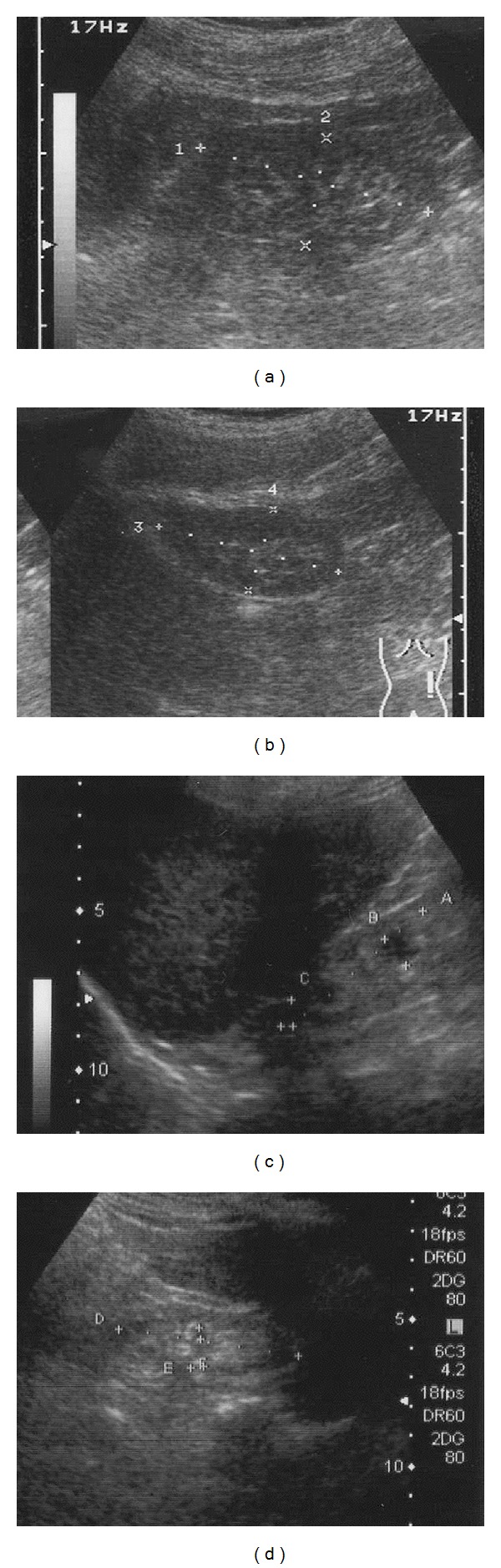
Example of sonographic measurements of the same patient. First measurement (a) right, (b) left, 4 years 9 months after the first measurement (c) right, (d) left.

Of 229 outpatient chronic hemodialysis patients, 109 patients, who had neither hereditary polycystic disease nor severe acquired cystic disease of kidney, were selected; we performed ultrasonography (US) twice or three times to measure their maximum renal diameter (mean of both kidneys), and the yearly alteration rate of the diameter was calculated. The US study was performed by a sole medical examiner. The largest caliber of the imaged kidney was measured using the measurement tool loaded in the US machine (Aroca SSD-280, Toshiba Nemio 30, Japan) (Figure 1). The US study of the kidney was mainly carried out intercostal method through ribs with 3.75 MHz linear probe. Some of the patient's data had been measured more than three times, and for those cases, the longest interval between the two data was used for calculation. The yearly alteration rate (cm/year) was calculated as follows: {(mean of both kidney diameter (KD1 − KD2 cm))/measurement interval (month)} × 12 (months). The results were studied to find out the factors that may influence the alteration rate of kidney diameter: age, gender, and the original disease (compared among three groups, chronic glomerulonephritis and IgA nephropathy, diabetes, and others including hypertension). To evaluate whether the alteration rate was stable all through the “on HD period” or not, patients were divided to nine groups according to the interval from the induction of HD to the first measurement. And the mean alteration rate of these nine groups were compared. All the patients included in this study were outpatient of the hemodialysis clinic. All patients agreed with signature that their clinical data or physical findings would be used for statistical study of the Japanese Society for Dialysis Therapy, and other clinical studies. This study did not violate ethical standards of the Declaration of Helsinki and its revisions, because the measurements were performed during the annual routine health check up for HD patients without any additional invasive procedures, and the purpose of the measurement was explained by the examiner and verbal allowance was obtained by the patient at the time of examination.

All values were expressed as mean ± standard error (SE). Statistical evaluations of various parameters were performed using Stat View 4.5 for Mac (ABACAS Concepts, Inc. CA USA), and each analytical method was indicated in the table. A *P* value of <0.05 was regarded as statistically significant.

## 3. Results

**Figure 2:**
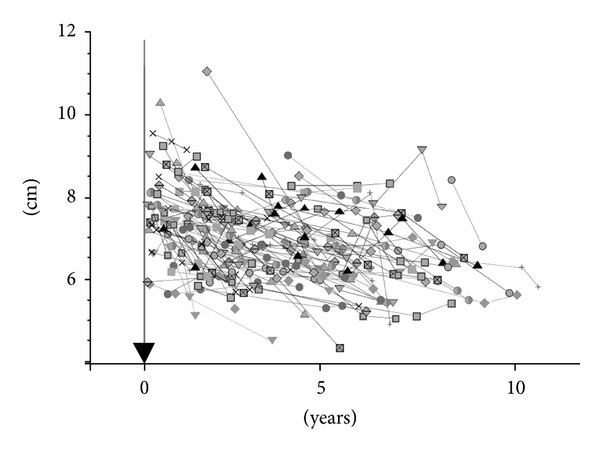
Measured raw data of 109 patients. Mean diameters of the both kidneys were plotted and measurements of the same patient were connected by line. (▼) Induction of hemodialysis. Lower axis represents duration of hemodialysis.

**Figure 3:**
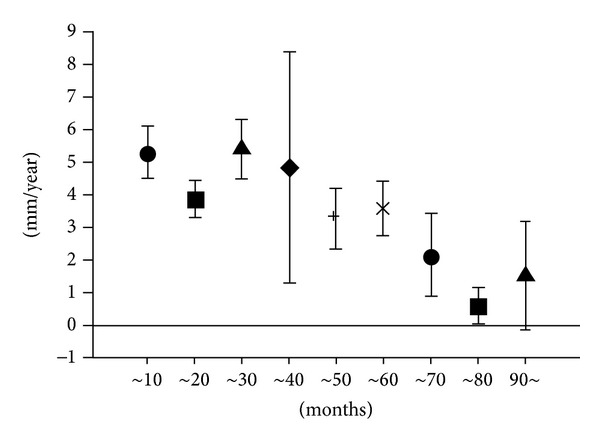
Decrease rate of the mean renal diameter and duration of hemodialysis. Samples were divided to 9 categories according to the time of the first measurement after induction of hemodialysis. The decrease rate was large when the first measurement was close to the induction of hemodialysis.

Raw data obtained by all 109 patient's measurements and connected in line between the same patient's data plots is indicated as in Figure 2. The average interval from the induction of HD to the first measurement was 29.5 ± 2.5 months (from 0.6 to 114 months), and the average interval of the two measurements was 35.9 ± 2.2 months (from 5.8 to 86 months). The average of all measured renal largest diameter was 6.91 ± 0.06 cm (4.3 to 11.1 cm). The average decrease rate of renal diameter was 4.34 ± 0.4 mm/year, which was simply obtained from the mean of 109 patients' results. Among the three original groups, CGN + IgA and diabetes groups were younger than HT + others group (*P* < 0.05), and diabetes group showed larger renal diameter at the first measurement (*P* < 0.05) as indicated in Table 1. However, no difference was seen on the decrease rate in relation to gender, age, and original disease (Tables 1 and 2). Comparing the samples those were divided to 9 categories according to the time of the first measurement after induction of HD, the decrease rate was large when the HD vintage was small, suggesting that the alteration rate reduced according to the duration of HD (5.3 ± 0.8 mm/year, *n* = 33, first measurement not more than 10 months after induction of HD, 3.9 ± 0.6 mm/year, *n* = 18, ~20 months, 5.4 ± 0.9 mm/year, *n* = 16, ~30 months, 4.9 ± 3.5 mm/year, *n* = 6, ~40 months, 3.3 ± 1.0 mm/year, *n* = 11, ~50 months, 3.6 ± 0.8 mm/year, *n* = 11, ~60 months, 2.1 ± 1.3 mm/year, *n* = 5, ~70 months, 0.6 ± 0.6 mm/year, *n* = 5, ~80 months, 1.5 ± 1.6 mm/year, *n* = 4, and ~80, *P* = 0.014, Kruskal-Wallis) as indicated in Figure 3.

## 4. Discussion

Although gradual decrease of the kidney size is frequently observed in HD patient, to the best of our knowledge, no studies have been found in the English medical literature reporting the actual decrease rate of the kidney size by measuring the same patient consecutively. Therefore, the decrease rate and the influence of original disease on the decrease speed, were poorly understood. Our study indicated that kidney size decreased approximately 4 mm per year in an average, and the decrease rate slowed as the dialysis vintage increased. Original disease, gender and age did not affect the decrease rate.

 In the Japanese literature, Uchida [2] studied on HD patients concerning kidney size, degree of cysts formation and possibility of cancer formation from the autopsy and surgical specimens. They reported that kidney size decrease the first three years after the induction of HD (Kidney size (cm) = *K* = 9.13 − 0.33*X* (years)), then, increased due to ACDK formation until 8 years (*K* = 5.43 + 0.15*X*), and after 8 years, kidney size decreased again (*K* = 7.62 − 0.58*X*). Yamaguchi et al. [3] reported from US observation, that kidney size decreased the first three years after the induction of HD, kept the same size until 8 years, and then start to increase size due to ACDK formation. According to their study, the renal shrink rate of patients who had diabetes was smaller than those who had other diseases in the first 5 years from induction of HD. These studies were not suitable to evaluate the common decrease rate of renal size after induction of HD, that in they include both cystic and noncystic kidneys [1–3]. We excluded 125 ACDK-formed patients out of 234 patients and evaluate 109 patients. 

 Our study did not consider the initial renal diameter at the induction of HD, as there was no data, and it would be better if the measurement interval had been regular to obtain more accurate data. The shrinkage of kidney is thought to not start at the induction of HD but at the histological change into end-stage kidney by original diseases. Other previous study reported that the renal diameter of the patients having diabetes was larger than that of those having other diseases such as CGN. Renal hypertrophy is thought to one of the signs of renal impairment of diabetic nephropathy [8, 9]. The decrease rate of renal diameter in patients who have diabetes is reported to be faster than in those who have other diseases in the beginning of HD [3]. In our study, the mean of the first measured renal diameter of the diabetic patients was also larger than that of other original diseases as indicated in the Table 2. However, according to our study, there were no difference in the decrease rate of renal size among the original diseases.

 Some cases showed different types of renal cortical shrinkage such as the increase of parapelvic adipose tissue, not altering total renal diameter so much. There were no malignant tumors in our study including ACDK case, though they were excluded from the calculation of renal size. Although more study will be needed to find out the relation between tumor formation and alteration of renal appearance, our study suggested that macroscopic change of end-stage kidney would be almost the same among the original diseases that were responsible for inducing HD.

## 5. Conclusion

Chronological change of renal size on chronic HD was studied for 109 HD patients using US examination consecutively. Renal diameter decreased approximately 4.3 mm each year, and the decrease rate slowed as the duration of hemodialysis increased. The decrease rate was not affected by the original disease, age, and gender.

## Figures and Tables

**Table 1:** Patient's backgrounds and renal size.

	CGN + IgA	Diabetes	HT + others	*P *
*n *	21	31	57	
Age (years)	54.7 ± 2.5	58.4 ± 1.8	62.4 ± 1.4	0.015
Mean renal diameter at the first measurement (cm)	7.07 ± 0.16	7.77 ± 0.19	7.20 ± 0.13	0.01
HD vintage to the first measurement (months)	35.2 ± 6.2	26.2 ± 4.0	29.1 ± 3.6	0.46
Measured interval to calculate decrease rate (months)	38.5 ± 4.6	30.0 ± 3.9	38.2 ± 3.1	0.23
Decrease rate (mm/year)	3.81 ± 0.98	4.74 ± 0.83	4.34 ± 0.50	0.72

CGN: chronic glomerulonephritis, IgA: IgA nephropathy, and HT: hypertension.

Each statistical difference was evaluated by ANOVA.

**Table 2:** Possible factors and the alteration rate of renal size.

Factors	Analysis	*P *	Influence
Age			
Average 59.6 ± 11.3 years	Correlation coefficient 0.01	Regression *P* = 0.91	No
Gender			
Male *n* = 59	Decrease rate 4.55 ± 0.57 mm/year	Student's *t*-test *P* = 0.46	No
Female *n* = 50	4.10 ± 0.56		
Disease			
CGN + IgA	Decrease rate 3.81 ± 0.98 mm/year	ANOVA *P* = 0.723	No
Diabetes	4.74 ± 0.83		
HT + others	4.34 ± 0.50		

CGN: chronic glomerulonephritis, IgA: IgA nephropathy, and HT: hypertension.
